# Swedish translation and linguistic validation of the multidimensional dyspnoea profile

**DOI:** 10.3402/ecrj.v3.32665

**Published:** 2016-11-08

**Authors:** Magnus Ekström, Josefin Sundh

**Affiliations:** 1 Division of Respiratory Medicine and Allergology, Department of Clinical Sciences, Lund University, Lund, Sweden; 2 Department of Respiratory Medicine, School of Medical Sciences, Örebro University, Örebro, Sweden

**Keywords:** dyspnoea, breathlessness, multidimensional, respiratory disease, heart disease, measurement, Swedish

## Abstract

**Background:**

Dyspnoea, the feeling of breathing discomfort, consists of multiple dimensions that can vary in intensity, including the level of unpleasantness, qualities or descriptors of the sensation, emotional responses, and impact on function. No validated instrument for multidimensional measurement of dyspnoea is available in Swedish. The Multidimensional Dyspnea Profile (MDP) was recently developed to measure the unpleasantness, sensory qualities, and emotional responses of dyspnoea across diseases and settings. We aimed to take forward a Swedish version of the MDP.

**Methods:**

Translation and linguistic validation of the MDP was conducted in collaboration with a specialised company in the field (Mapi, Lyon, France). The structured process involved forward and backward translations by two independent certified translators, input from an in-country linguistic consultant, the developers, and three respiratory physicians. Understandability and acceptability were evaluated through in-depth interviews with five patients with dyspnoea in accordance with international guidelines.

**Results and Conclusion:**

A Swedish version of the MDP was obtained and linguistically validated. The MDP includes 11 rated items: the immediate unpleasantness of the sensation, the presence and intensity of five sensory qualities, and the intensity of five emotional responses to dyspnoea. The time period of measurement is specified by the user. The MDP is copyrighted by the developers but can be used free of charge in the context of non-funded academic research.

**Conclusion:**

The MDP is the first instrument for measuring multiple dimensions of dyspnoea available in Swedish and should be validated across diseases and settings. Multidimensional measurement is essential for improved assessment and management of dyspnoea in research and clinical care.

Dyspnoea (or breathlessness), the subjective feeling of breathing discomfort, is a cardinal symptom of heart and lung disease (1). The prevalence of dyspnoea is high among middle-aged and elderly in the population (2) and increases steeply with increasing disease severity across a range of underlying conditions (3).

Dyspnoea has strong adverse effects on health outcomes. It is linked to reduced physical activity, worsening deconditioning, increased anxiety and depression, impaired quality of life, loss of the will to live near death, increased risk of hospitalisation, and earlier death (1, 4). The importance of dyspnoea has been highlighted in recent years, and dyspnoea has been included in the evaluation of disease severity (5) and prognosis in patients with chronic obstructive pulmonary disease (COPD) (5). Dyspnoea is in fact a stronger predictor of mortality than the level of airflow limitation in COPD (6). Dyspnoea is a negative prognostic factor across severities of heart failure (7). In patients with suspected heart disease undergoing cardiac stress testing, more severe dyspnoea is a strong risk factor for earlier death from cardiac disease and for earlier death overall (8).

Dyspnoea consists of several different qualitatively distinct sensations that vary in intensity (1). Several dimensions of this symptom can be differentiated by the individual: the experienced *intensity* and *unpleasantness*, the associated *emotional response*, and the *functional impact* on the person's life (1).

Despite its serious impact, dyspnoea remains frequently underreported, unmeasured, and undertreated in clinical practice (9). Unpleasantness, emotional responses, and the sensory qualities have been measured in different studies using varying (disease-specific) scales, wordings, and time frames (1, 10). This makes it difficult to compare findings between studies, patient populations, and settings. Importantly, standardised multidimensional measurement is essential to adequately capture treatment effects in clinical trials. For example, opioids have been found to have a stronger effect on the unpleasantness and associated anxiety than on the intensity of dyspnoea (11), and pulmonary rehabilitation improves the patient's coping and function in relation to dyspnoea whereas the symptom intensity may remain unchanged (12).

The Multidimensional Dyspnea Profile (MDP) is a recently developed tool to separately measure the immediate breathing discomfort, five sensory qualities, and five emotional responses of dyspnoea across underlying disease and (laboratory and non-laboratory) settings (13, 14). The time frame or situation of the measurement is defined by the user. The MDP was published by Banzett et al. (10) and can be used free of charge in the context of not-funded academic research. Distribution fee will apply in the context of funded academic and commercial use. It has been translated and used in several languages including French for France, French for Belgium, French for Canada, German, Dutch for Belgium, Dutch for the Netherlands, English for the Canada, English for the UK (10, 15).

There is currently no tool for multidimensional measurement of dyspnoea available in Swedish. A Swedish version of MDP could facilitate improved detection and measurement in research and clinical practice in Sweden, as well as comparisons of dyspnoea across languages.

We therefore aimed to develop a linguistically validated Swedish translation of the MDP.

## Methods

Structured translation and linguistic validation of the MDP (10) from the original American English into Swedish was conducted in collaboration with a company (Mapi SAS, Language Services Unit, Lyon, France) specialised in translation and linguistic validation of patient-reported outcome measures. The MDP was used in this project with the permission of the copyright holder, Robert B. Banzett, USA.

### Ethical considerations

The study was approved by the regional ethics committee at Lund University (DNr: 2016/16). Written informed consent was not required as no personal data on participants were collected.

### Translation

Translation and linguistic validation was conducted in a structured, multistage process according to international guidelines (16, 17).

After permission to translate, the MDP was obtained from the developer (10), and the original instrument was forward translated into Swedish independently by two certified translators. The forward translations were analysed and reconciled by an in-country consultant. Clarifications and information were obtained from the developers throughout the whole translation process. Quality control by Mapi Language Services established a translation version 1, which was back translated into the original language (American English). Comparison of the backward translation to the original instrument conducted by the in-country consultant as well as review by the developer resulted in a translation version 2.

### Clinicians’ review

The translation version 2 was then reviewed by three Swedish specialists in internal and respiratory medicine (authors ME and JS, as well as a colleague currently working in palliative medicine), who provided detailed feedback on the understandability and validity of key concepts to users of the instrument and people with dyspnoea. The translation was revised by Mapi Language Services with input from the in-country consultant and the developer, resulting in version 3 of the translation.

### Patient interviews

The translation version three was evaluated in individual in-depth, cognitive interviews with five Swedish patients with dyspnoea to investigate its clarity, understandability, and acceptability. Patients were recruited by Mapi Language Services, led by the in-country consultant. The participants commented on their understanding of each item and suggested alternative formulations for problematic wordings. After summarising, revising, and proofreading, a final linguistically validated translation was issued.

## Results

Revisions were made to the initial translations, especially regarding the wording of the descriptive items (second domain) after extensive input from the clinicians’ review, the in-country linguistic consultant, and the developers.

The copy of the final certified, linguistically validated Swedish translation of the MDP is found in Supplementary file for review purpose only.

## Discussion

This project has taken forward a linguistically validated Swedish translation of the MDP, the first tool for multidimensional measurement of dyspnoea in Swedish. The MDP is developed for measurement across diseases. The translation is made available for independent validation in people with different underlying diseases, such as obstructive pulmonary disease, interstitial lung disease, cardiovascular disease, hypoventilation disorders, and other conditions causing dyspnoea, and for validation across laboratory and non-laboratory settings.

The translation was conducted by specialists in the field (Mapi) in accordance with international guidelines for patient-reported outcomes to be used as outcomes in interventional trials (16, 17).

The MDP builds on extensive mechanistic studies of multidimensional pain and dyspnoea (10). The original American version was validated in response to laboratory stimuli (10) and in 151 patients admitted to an emergency department for acute dyspnoea (29% had asthma, 27% COPD, 19% pneumonia, 13% heart failure, and 13% other) (13).

### Use of the MDP

The MDP can be administered by an investigator/healthcare provider or be self-completed with a person on hand to answer questions during initial administration (10). The time frame or situation of the measurement is defined by the user (10). Before use, it is important that the subject receives standardised information and instructions as described elsewhere (10), for reliable and valid measurement. The MDP consists of 11 items divided into three domains (10). In the first domain, the unpleasantness or discomfort of the breathing sensation is rated on a numerical rating scale (NRS) between 0 (‘neutral’) and 10 (‘unbearable’). In the second domain, the subject first indicates which of five descriptions that match their breathing discomfort and indicates the most accurate descriptor. The subject then rates the intensity of each descriptor (and of another self-specified sensation if needed) on an NRS between 0 (‘none’) and 10 (‘as intense as I can imagine’). In the third domain, the subject rates the intensity of emotional responses to their breathing discomfort (depression, anxiety, frustration, anger, and fright) on an NRS between 0 (‘none’) and 10 (‘the most I can imagine’) (10).

The MDP was completed within a few minutes by most participants (10). The MDP and all its derivative works such as translations are copyrighted by the developers. The original MDP and all its translations are distributed by the Mapi Research Trust (https://eprovide.mapi-trust.org), which should be contacted for any enquiry about the questionnaire and the requirements regarding its use. The original reference (10) should be cited by all papers using the MDP. An advantage of the MDP is that scores can be analysed for each scale or as the mean of each domain (immediate perception or emotional response domain) separately (10).

A study is underway to validate the MDP in patients with cardiopulmonary disease in Swedish outpatient clinics. Further work is needed – including in laboratory and in-patient settings and in people suffering from – including determination of the clinical, minimally important difference for different dimensions of dyspnoea for use in clinical trials (18). In conclusion, the Swedish version of the MDP to measure the unpleasantness, qualities, and emotional responses to dyspnoea is now available for validation across patient populations and settings. Standardised multidimensional symptom measurement using MDP could be of fundamental importance for improved research and clinical care of patients suffering from dyspnoea.

## Supplementary Material

Swedish translation and linguistic validation of the multidimensional dyspnoea profile
