# Supplementary material for: Swedish translation and linguistic validation of the multidimensional dyspnoea profile
Source: Eur Clin Respir J. 2016 Nov 8;3:10.3402/ecrj.v3.32665. doi: 10.3402/ecrj.v3.32665 (PMC5103669; doi:10.3402/ecrj.v3.32665)
Supplement: Swedish translation and linguistic validation of the multidimensional dyspnoea profile [file ECRJ-3-32665-s001.pdf]

Appendix 1. Sample Copy of the Swedish version of the Multidimensional Dyspnea Profile (MPD) for review purpose only. Do not use without permission from Mapi Research Trust (https://eprovide.mapi-trust.org).

MULTIDIMENSIONELL DYSPNÉPROFIL ©2011 R.B. Banzett. All rights reserved.

Text att läsa första gången:

Syftet med det här frågeformuläret är att hjälpa oss förstå hur du upplever din andning.

Det finns inga svar som är rätt eller fel. Vi är intresserade av vad du kan berätta om din egen andning.

På den här sidan ber vi dig att berätta hur obehaglig din andning känns. På en senare sida kommer vi att fråga hur intensiva eller starka dina andningsupplevelser är. Skillnaden mellan de här två aspekterna i dina andningsupplevelser kan bli tydligare om du tänker dig att du hör ett ljud, till exempel från en radio. När ljudvolymen ökar kan jag fråga hur starkt det låter eller hur obehagligt det är att höra ljudet. Till exempel kan musik som du hatar vara obehaglig även vid en låg volym och bli ännu obehagligare när volymen höjs; musik som du tycker om blir inte obehaglig ens när volymen höjs.

Skala A1

Använd den här skalan för att gradera hur **obehagliga eller besvärliga** dina andningsupplevelser är [var], hur **svår** din andning känns [kändes].

Fokusera på perioden när___________________

←	←	0	1	2	3	4	5	6	7	8	9	10
BEHAGLIG		NEUTRAL										OLIDLIG

Sample copy – Do not use without permission

SQ (Sensorisk kvalitet) val

Nedan följer meningar eller beskrivningar som delats in i meningar med liknande betydelse.

Steg 1: Sätt ett kryss för varje mening som innehåller ord som beskriver hur din andning känns [kändes] under __________________ (ange fokusperioden).

Steg 2: Sätt ett kryss även för *den* mening som bäst beskriver hur din andning känns [kändes].

	Steg 1		Steg 2
Om *NÅGON* av beskrivningarna i meningen stämmer in på dig, välj den meningen.	STÄMMER INTE	STÄMMER	BESKRIVER BÄST
Min andning kräver muskelarbete ***eller*** ansträngning.			
Jag får inte tillräckligt med luft ***eller*** jag kvävs ***eller*** jag vill ha mer luft.			
Min bröstkorg och mina lungor känns trånga ***eller*** sammantryckta.			
Min andning kräver mental ansträngning ***eller*** koncentration.			
Jag andas mycket.			

Sample copy – Do not use without permission

SQ (Sensorisk kvalitet) skalor

Använd följande skalor för att gradera intensiteten på de andningsupplevelser som du har [hade]. Jämför med volymen på ett ljud som oavsett om det är behagligt eller ett obehagligt kan vara en stark upplevelse utan att vara obehagligt.

Fokusera på perioden när ______________________

Om NÅGOT av orden i meningen stämmer in på dig ska du gradera meningen.	**INTE ALLS**										**DEN STÖRSTA INTENSITET SOM JAG KAN FÖRESTÄLLA MIG**
Min andning kräver muskelarbete ***eller*** ansträngning.	0	1	2	3	4	5	6	7	8	9	10
Jag får inte tillräckligt med luft ***eller*** jag kvävs ***eller*** jag vill ha mer luft.	0	1	2	3	4	5	6	7	8	9	10
Min bröstkorg och mina lungor känns trånga ***eller*** sammantryckta.	0	1	2	3	4	5	6	7	8	9	10
Min andning kräver mental ansträngning ***eller*** koncentration.	0	1	2	3	4	5	6	7	8	9	10
Jag andas mycket.	0	1	2	3	4	5	6	7	8	9	10
Annat*	0	1	2	3	4	5	6	7	8	9	10

*Om det behövs kan du lägga till ytterligare beskrivningar av dina andningsupplevelser.

Sample copy – Do not use without permission

A2-skalor

När din andning inte känns som den brukar kan det hända att du upplever vissa ”känslor”. Använd skalorna nedan till att förklara hur dina andningsupplevelser har fått dig att känna dig – sätt noll på de känslor du inte har känt.

Fokusera på dina känslor under perioden när ______________________

	**INTE ALLS**										**MESTA TÄNKBARA**
Deprimerad	0	1	2	3	4	5	6	7	8	9	10
Ängslig	0	1	2	3	4	5	6	7	8	9	10
Frustrerad	0	1	2	3	4	5	6	7	8	9	10
Arg	0	1	2	3	4	5	6	7	8	9	10
Rädd	0	1	2	3	4	5	6	7	8	9	10
Annat?	0	1	2	3	4	5	6	7	8	9	10

Sample copy – Do not use without permission
